# Standardized clinical assessment and management plans revisited: lessons learned from a decade of implementation

**DOI:** 10.1093/haschl/qxag036

**Published:** 2026-02-10

**Authors:** Michael Farias, Peta Alexander, Jeffrey Geppert, Paige Glavin, Jessily Ramirez-Mendoza, Paul Casale, Kathy Jenkins

**Affiliations:** Department of Cardiology, Boston Children's Hospital and Department of Pediatrics, Harvard Medical School, Boston, MA 02115, United States; Department of Cardiology, Boston Children's Hospital and Department of Pediatrics, Harvard Medical School, Boston, MA 02115, United States; Health Business Unit, Battelle Memorial Institute, Columbus, OH 43201, United States; Health Affairs, Boston Children's Hospital, Boston, MA 02115, United States; Health Affairs, Boston Children's Hospital, Boston, MA 02115, United States; Departments of Medicine and Population Health Sciences, Weill Cornell Medical College, New York, NY 10065, United States; Department of Cardiology, Boston Children's Hospital and Department of Pediatrics, Harvard Medical School, Boston, MA 02115, United States

**Keywords:** standardized clinical assessment and management plans (SCAMPs), clinical decision support, artificial intelligence in health care, health information technology, care standardization, learning health systems, value-based care

## Abstract

**Introduction:**

Originally introduced in *Health Affairs* in 2013, Standardized Clinical Assessment and Management Plans (SCAMPs) are clinician-developed, modifiable care pathways designed to reduce unwarranted variation and optimize resource use while preserving professional judgment. Unlike traditional clinical practice guidelines that prescribe “best” practice, SCAMPs begin with consensus-based “sound” practice and emphasize iterative learning from real-world deviations and outcomes. Initially developed for conditions with limited evidence, SCAMPs have since expanded across a wide range of diagnoses and care settings.

**Methods:**

We conducted a structured historical review of more than 40 peer-reviewed publications describing SCAMPs development, implementation, evaluation, and iterative refinement. The review synthesizes reported experiences across clinical domains, settings, and study designs, and is intended as a descriptive, perspective-oriented assessment rather than a formal systematic review.

**Results:**

Published SCAMPs reports describe broad deployment across diverse conditions and institutions, with recurrent findings of reduced practice variation, changes in resource utilization, iterative pathway refinement, and high reported adherence among participating clinicians. The literature also reflects important limitations, including heterogeneity of study designs, limited evaluation of harms, equity, patient-reported outcomes, and implementation burden, and likely underrepresentation of unsuccessful implementations. Early reliance on paper-based workflows constrained scalability and consistency of use.

**Conclusion:**

This perspective synthesizes the published SCAMPs experience, highlighting reported benefits alongside implementation conditions, risks, and limitations. SCAMPs are best understood as a clinician-led methodology whose value is conditional on governance, analytic capacity, patient safety oversight, and attention to equity. Emerging informatics standards and artificial intelligence tools may enhance scalability and learning, but require careful governance to avoid amplifying bias or harm.

## Introduction

In the May 2013 edition of *Health Affairs*, the authors proposed the Standardized Clinical Assessment and Management Plans (SCAMPs) methodology as a general framework for addressing the persistent challenges of evidence generation, standardization of practice, improvement in health outcomes, and reduction in avoidable utilization.^1^ Twelve years later, SCAMPs continues to be a robust framework for addressing these challenges across a broad range of clinical domains.^2,3^ Moreover, advances in electronic health records (EHRs), interoperability, and artificial intelligence (AI) make SCAMPs implementation potentially scalable and reproducible across the health care system. Such systematic adoption of SCAMPs by health plans would potentially provide a “self-correcting” solution to prevailing policy concerns about prior authorization and up-coding.^4^

This paper reviews the original justification for SCAMPs, the lessons learned from more than 40 peer-reviewed studies of SCAMPs implementation, the manner in which Health Information Technology (Health IT) supports the modernization of SCAMPs, and the application of SCAMPs to health care payment reform.

SCAMPs should be understood as a methodology rather than a discrete clinical tool. Like other quality improvement approaches, SCAMPs comprise a set of core functions—explicit acknowledgment of uncertainty, consensus-based initial pathways, structured capture of positive deviations, prospective data collection, and iterative multidisciplinary review—while allowing flexibility in local implementation. Their value is therefore conditional, depending on governance, analytic capacity, safety oversight, and attention to equality, rather than inherent to the approach itself.

## Background and significance

Clinical practice guidelines (CPGs), often informed by robust evidence from randomized control trials (RCTs), attempt to define best practice for a particular medical diagnosis and are used to inform and standardize clinical practice.^5,6^ CPGs, however, can be challenging to create for certain areas of medicine where high-quality evidence is lacking, can become quickly out of date, can lack generalizability to a diverse patient population, can be subject to undue influence from industry, and can erode physician autonomy in clinical decision-making.^7-12^ In an effort to combat these concerns, SCAMPs were introduced in 2009 as a clinician-designed alternative to promoting care standardization, improving care delivery, and reducing unnecessary resource utilization.^1,13^

Each SCAMP targets a heterogeneous patient population with a single underlying diagnosis or chief complaint, providing a clinician with a consensus-based expert guideline even for diagnoses where limited data exist. A unique and central tenet of SCAMP methodology is an emphasis on targeted data collection followed by rapid and iterative improvement to achieve optimization. Rather than attempt to define a “best” practice like a CPG does, a SCAMP begins with “sound” practice and employs clinician feedback and learning mechanisms informed by diversions from the “sound” pathway to improve and promote positive, innovative changes to the clinical care of each condition.

## What is a SCAMP and how does it work?

SCAMPs are a quality improvement initiative designed specifically for areas of medicine where uncertainty in clinical decision-making exists. Conditions for which limited data on management and outcomes exist are ideal targets for SCAMPs; however, even for conditions where high-quality evidence is available, SCAMPs can still provide value if significant practice variation exists. Importantly, SCAMP methodology is applicable to a wide range of medical diagnoses and can be employed in outpatient, inpatient, and even procedural settings. Examples of the range of SCAMPs are listed in Table 1.

**Table 1.** Examples of various SCAMP target diagnoses.

Target Diagnoses	Target Diagnoses
Acute kidney injury	Catheterization for aortic stenosis
Congenital diaphragmatic hernia	Distal radius fracture
Fetal congenital heart disease	Growth failure
Ileocolic intussusception	Immune thrombocytopenia
Influenza and pneumonia	Pediatric chest pain
Postoperative pain	Reconstructive breast surgery
Severe acute asthma	Urinary incontinence

Source: Author's analysis of SCAMPs manuscripts (Appendix 1).

Once a target area for a SCAMP is chosen, the original SCAMP design and implementation process is as follows:

- A multidisciplinary expert committee is gathered with stakeholders including providers, nurses, therapists, pharmacists, information technology experts, safety and quality officers, and patients and families if appropriate.
- A background position paper is composed to review the highest-quality and most up-to-date evidence available on the condition, and to review any existing guidelines.
- A list of “plausible outcomes,” or specified statements about diagnostic or therapeutic approaches that are *potentially refutable* by accumulating and reviewing unbiased follow-up data, is generated. This become the focus of targeted data collection in the SCAMP.
- A management pathway is designed to capture consensus-based “sound” practice. The SCAMP-managed episode of care, or SMEOC, is defined and can vary from short episodes such as single clinic visits or procedures, to longer episodes such as long-term management of a chronic condition. For each encounter within the SMEOC, a SCAMP data form is composed not only to include the care pathway, but also to collect targeted data elements and to capture clinical reasoning for any diversion from the pathway's recommendations.
- The SCAMP is implemented by supplying the SCAMP data forms to clinicians at the point of care. Data are collected prospectively on the form and care diversions from the pathway are encouraged, with real-time capture of these diversions along with the rationale for the diversion. This allows for both preservation of physician autonomy and learning from diversions as a source of innovation and potential pathway improvement.^14^
- The data generated from SCAMPs are continuously gathered and periodically reviewed by statisticians before being presented to the expert committee. Data gathered include information on pathway adherence, targeted assessment of plausible outcomes, and reasons for pathway diversions.
- Semiannual or more frequent review of the SCAMP is completed by the expert committee. Data collected may support or refute plausible outcomes. Adjudication of pathway diversions can lead to valuable insights on the quality of SCAMP recommendations. Furthermore, consistent SCAMP nonadherence for certain patient groups may lead to an understanding of whether an alternative pathway could lead to improved outcomes for that patient group.
- Based on this review and ongoing review of the literature, the SCAMP undergoes progressive modification to incorporate findings and iteratively improve.

**Figure 1.**
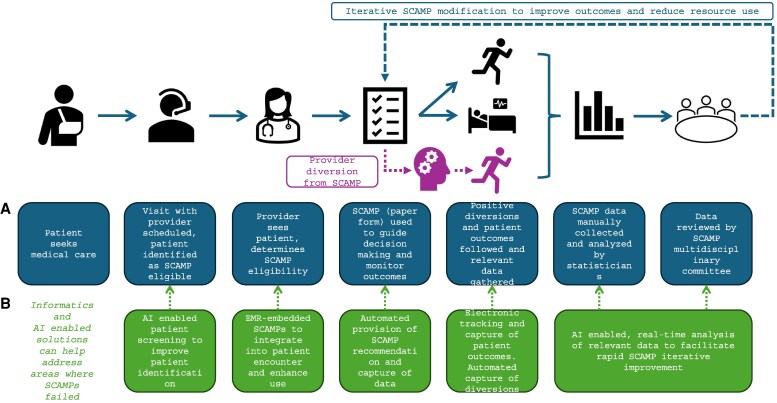
Overview of the SCAMP implementation with informatics and AI integration opportunities. Source: Author's analysis; Notes: (A) When a patient seeks medical care the patient is manually identified as eligible for a particular SCAMP. (B) At each step, informatics and AI-enabled solutions could work to significant enhance adoption and the quality of the SCAMPs process.

Figure 1 offers a schematic of SCAMP implementation, including collection of data, diversions, and iterative improvement cycles.

## The impact of SCAMPs

Implementation of over 60 SCAMPs has yielded valuable insight into the successes and failures of the process.^15^ We conducted a structured historical review of peer-reviewed SCAMPs publications identified through searches using the terms “Standardized Clinical Assessment and Management Plan(s)” and “SCAMP,” supplemented by reference review. Articles were included if they described SCAMP design, implementation, evaluation, or iterative modification, and excluded if the acronym was used for unrelated concepts.

This review incorporates elements of PRISMA guidance for transparency but was not designed as a formal systematic review. The resulting literature is heterogeneous, dominated by pragmatic quality improvement studies from early adopter institutions, and likely underrepresents null, unsuccessful, or harmful implementations. Findings should therefore be interpreted as descriptive and hypothesis-generating rather than definitive. A list of all published articles reviewed is included in Appendix 1; highlights of the learnings are discussed here.

### Applicability

SCAMPs can be developed locally by experts in delivering care in a particular setting or patient population, or can be developed broadly for assessing and managing conditions across a range of health care environments. SCAMPs have been used in over a dozen health care systems ranging from standalone hospitals in rural settings to large academic medical centers, and have also been used across two professional societies.^1^ Studies on SCAMP implementation demonstrate the effectiveness of the process to inform both specific and more general areas of practice (Appendix 1). Originally designed using paper forms, one early challenge to adoption was the lack of a robust informatics solution to implement SCAMPs and readily capture and analyze data. Artificial intelligence (AI) applications are now being explored to develop and implement SCAMPs electronically, leveraging the Electronic Health Record (EHR) and embedding into routine clinical workflow to automatically gather relevant data and inform the SCAMPs process.^16^

### Pathway improvement

Studies on SCAMP implementation frequently mention the iterative nature of the improvement. For example, a SCAMP for patients with critical status asthmaticus demonstrated that three cycles of improvement led to iterative changes that reduced resource use and hospital length of stay without compromising outcomes.^17^ It is an expectation that over time, most SCAMPs will be modified to reduce resource utilization as testing modalities that are found to be low yield are eliminated from the care delivery algorithm or focused on a smaller group of patients. A SCAMP on pediatric chest pain helped lead to identification of exertional symptoms as one of the primary indications for obtaining an echocardiogram, and focusing recommendation of this test to those patients with red-flag symptoms led to both a decrease in unnecessary echocardiograms performed and an increase in the number of appropriate echocardiograms.^18,19^ In a SCAMP on patients with a dilated aorta, refinement of a recommendation to refer to a genetic specialist only in situations where concerning signs, symptoms, or family history were present led to increased clinician buy-in and improved adherence rates from 20% to 75%.^1^

### Practice variation

Medicine is faced with the challenge of controlling increasing costs while being able to maintain safe, high-quality, and equitable standards of care. Variation in clinical practice leads to increased costs without improvement in outcomes.^20^ SCAMPs, as previously mentioned, are well-suited for areas in which clinical uncertainty and thus high practice variation exist. For instance, an analysis on decisions made by pediatric cardiologists showed that less than 20% of decisions were made based on data from published research studies and almost 15% of decisions were deemed arbitrary in nature.^21^

Implemented SCAMPs have been shown to lead to recommendation adherence rates that exceed 80%, thus reducing practice variation.^1^ Importantly, when a recommendation is being poorly followed, the SCAMP can help to rapidly identify the reasons why, and then allow for mediation. Adjudication of diversions from SCAMPs showed about half of diversions to be non-justified, and most commonly related to arbitrary physician preference, non-justifiable patient or family preferences, or poor documentation.^14^ When a SCAMP for patients with hypertrophic cardiomyopathy was failing to standardize follow-up intervals, leading to more frequent than recommended clinical visits and testing, this was discovered to be related to parental and provider anxiety in the setting of known genetic mutations.^1^ Provider education on expected disease progression led to safe lengthening of follow-up intervals.

### Resource use

To mitigate the growth of cost controlling methods such as prior authorizations and reimbursement reduction, it becomes imperative to ensure that unnecessary and under-necessary care and resource utilization are avoided. In the alternate, necessary resource use must also be ensured to promote positive outcomes. SCAMPs work well to attain this optimization of resource use through multiple avenues.

Low-probability testing*—*Diagnostic testing that is ordered despite being low-yield and potentially expensive carries with it the risk of incidental findings, false positive or negative results, and ineffective interpretation. Prior authorizations are a mechanism to try to reduce the use of such testing, but create a paperwork and administrative burden that can lead to provider frustration and inadvertent reductions in necessary testing. SCAMPs focus diagnostic testing for most patients on necessary and high-yield testing. A SCAMP on distal radius fractures worked to decrease imaging use and reduce other ancillary tests, thus helping to reduce unneeded testing and unnecessary return visits.^22^ Another SCAMP designed to reduce testing for adults presenting to the emergency room with chest pain considered to be low-risk showed that adherence to the SCAMP led to fewer unnecessary troponin checks and stress tests, in addition to shortening length of stay for those patients from 22 to 9 hours.^23^

High-cost therapeutics—Certain expensive medications and interventions that should be reserved for very specific clinical situations are an additional target of prior authorizations and reimbursement reductions or denials. SCAMPs have demonstrated more judicious use of these resources in multiple settings. In the aforementioned status asthmaticus SCAMP, mean duration of continuous albuterol therapy in the intensive care setting decreased from 24.9 to 17.5 hours.^17^ In addition, while initial standardization in the SCAMP led to an increase in the adjunctive use of Heliox, a gas mixture of helium and oxygen gas, from 22% to 60%, iterations of the SCAMP led to a decrease in use of this therapy to 14% without adversely affecting outcomes.

Low-probability referrals—Visits to specialists that have a low likelihood of yielding meaningful additional information often result from unfamiliarity on the part of the referring provider or anxiety from the patient or family about a possible condition. Furthermore, these referrals result in resource use that is largely wasteful. A SCAMP on pediatric chest pain, for example, helped to empower physicians to recognize red flags for referrals and to effectively communicate and negotiate with patients surrounding this emotionally laden complaint; furthermore, dissemination of these benefits to the primary care setting to prevent low-probability chest pain referrals was projected to lead to significant cost savings without missing significant disease.^24,25^

Resource substitution—The use of less expensive resource substitution, such as a visit with a nurse practitioner instead of a medical doctor or at a satellite site rather than hospital setting, can be suggested by a SCAMP to avoid using the most expensive resources without compromising outcomes. Serum-specific IgE and skin prick testing are tools used to test children for food allergies, but are imperfect predictors of whether a child will be at risk for a severe reaction during an oral challenge. A food allergy SCAMP set and continually refined serum and skin testing recommendations, allowing allergists to better determine risk and more safely triage patients to the appropriate setting for an oral food challenge—either an outpatient clinic setting if low risk or a higher resource infusion center if higher risk.^26^

### Outcome improvement

One principle of SCAMPs is that through care standardization, quality of care and clinical outcomes can be improved. In the aforementioned SCAMP on pediatric chest pain, wide variation in testing ordered for evaluation was found to be related to provider volumes and experience.^18,19^ Supporting providers with expert consensus and evidence through the SCAMP help increase the appropriateness of testing ordered; un- and under-necessary testing was reduced while the sensitivity of evaluation was increased. Another SCAMP used to manage patients with aortic stenosis undergoing catheter-based balloon angioplasty increased the frequency of “ideal” outcomes from 40% to 69% and reduced “inadequate” outcomes from 30% to 9% at a single institution.^27^

An additional important feature of SCAMPs is the ability to identify opportunities for further studies, such as a decision-point with enough uncertainty to inform the design of an RCT. There is also potential that the data collection embedded in a SCAMP can itself generate high quality data that can inform practice by evaluating the predefined plausible outcomes.

### Clinician engagement

CPGs can be criticized for their rigidity and lack of flexibility in provision of personalized patient care. This can make certain guidelines difficult to incorporate into practice workflow and can make them a source of clinician dissatisfaction due to a perceived erosion of autonomy.^7-9^ The SCAMPs process obtains clinician buy-in by engaging clinicians in all aspects of the design and implementation process, and as importantly, SCAMPs preserve provider autonomy and value their clinical acumen by welcoming diversions from the SCAMP pathway. By starting with “sound” practice the SCAMP acknowledges that it is imperfect, and clinicians recognize that the ability to divert from recommendations allows them to provide individualized care and contribute data that could lead to refinement of the SCAMP algorithm. In a survey, clinicians reported a preference of SCAMPs over traditional CPGs or other guidelines, and over 60% reporting being positive or highly positive in their evaluation of SCAMPs.^28^

Beyond standardization, SCAMPs can function as cognitive supports in areas of clinical uncertainty. By making expert consensus explicit, structuring decision points, and legitimizing evidence-informed restraint, SCAMPs may reduce cognitive load and enhance diagnostic confidence. Reported clinician buy-in appears driven not only by preserved autonomy, but by reduced decisional uncertainty. These benefits are not automatic and depend on thoughtful design and workflow integration.

### Fostering innovation

Most guidelines lack the ability to collect information on their use or disuse. This latter point is especially important—when a clinician does not feel that a guideline is applicable to a specific patient, the disuse of the guideline and the treatment of the patient as an “outlier” limits the capture of information on the care of that patient as a potential source of innovation. By not only permitting diversions but also capturing the rationale behind said diversion, SCAMPs acknowledge that some patients are so unique that assessment and treatment plans cannot be predefined, and SCAMPs have the ability to capture this knowledge.^29^ The identification of “positive diversions”—a concept based on the notion that local clinician acumen may have already solved some of the most difficult challenges in the care of a particular patient population—is a valuable source of information and innovation. Careful adjudication and analysis of diversions is thus an important step in effective modification and improvement of the SCAMP.^14^

## Implementation success factors and resource dependencies

Successful SCAMPs implementations share several enabling conditions. These include multidisciplinary clinical leadership with decision-making authority; analytic expertise to assess outcomes, variation, and safety signals; protected time for peer review and pathway revision; and operational support to integrate SCAMPs into routine clinical workflow.

While the form of these elements varies across sites, their function is consistent. Resource limitations—particularly analytic capacity, care coordination support, and informatics infrastructure—represent significant barriers and help explain variability in uptake and effectiveness. In settings lacking these capabilities, SCAMPs may stall, generate limited learning, or fail to deliver net benefit.

## Risks and governance

SCAMPs carry meaningful risks and limitations. Poorly governed SCAMPs may reify local practice patterns, reinforce bias, legitimize inefficient resource use, or delay recognition of harm. Reliance on clinician-driven identification and paper-based documentation can result in missed eligible patients, uneven participation, and selective uptake.

The published literature likely reflects positivity bias, as unsuccessful or abandoned SCAMPs are rarely reported. Most studies lack concurrent controls, formal assessment of implementation fidelity, or systematic evaluation of burden, equality, or adverse effects. As a result, reported benefits should be interpreted cautiously and not assumed to generalize across conditions, institutions, or health systems.

Claims that SCAMPs are “self-correcting” are conditional; without timely data, analytic capacity, and escalation authority, learning cycles may lag behind real-world harm or entrench suboptimal practice.

Like all clinical decision-support processes, SCAMPs may embed patient safety hazards if underlying assumptions, logic, or data inputs are flawed. Examples include incorrect dosing, omitted contraindications, or failure to account for age or comorbidity. Safe SCAMPs deployment requires linkage to institutional safety reporting, pharmacy review, and quality governance, with clear authority to suspend or modify pathways when safety signals or patient harm are identified.

Standardized pathways risk embedding structural and social bias if recommendations implicitly assume access to transportation, schedule flexibility, stable housing, or caregiving support. SCAMPs that recommend frequent follow-up or testing without accounting for these constraints may exacerbate inequalities.

High-quality SCAMPs therefore require explicit attention to social context, including participation by social workers and care coordinators. Deviations should be interpreted not solely as nonadherence, but often as signals of structural infeasibility that warrant pathway adaptation.

## Future directions

### Informatics-enabled SCAMPs: a roadmap

A significant shortcoming of the initial rollout of SCAMPs was the absence of interoperable, standards-based digital infrastructure, and clinical decision support tools which could facilitate SCAMP adoption, incorporation into the medical record, rapid data collection, and analysis for iterative improvement (Figure 1). The transition from paper-based SCAMPs to electronic SCAMPs would initially require a digitization phase of integration into the EHR, structured data collection, and electronic SCAMP forms (Table 2). Following digitization, an automation phase would include data extraction, diversion capture, and preliminary recommendation logic. Subsequent phases would include AI augmentation and learning health system integration.

**Table 2.** Informatics priorities for SCAMPs.

Priority	Informatics enabler
Identify SCAMP-eligible patients	EHR-based cohort discovery tools
Guide clinicians at point of care	CDS Hooks, SMART on FHIR apps
Capture deviations and rationale	Structured EHR inputs, NLP
Analyze outcomes and iteratively improve pathways	AI-based causal inference, dashboards
Share updated pathways	FHIR PlanDefinition updates across sites

Source: Author's analysis.

Abbreviations: AI, Artificial Intelligence; CDS, Clinical Decision Support; EHR, Electronic Health Record; FHIR, Fast Healthcare Interoperability Resources; NLP, Natural Language Processing; SCAMP, Standardized Clinical Assessment and Management Plan; SMART, Substitutable Medical Applications and Reusable Technologies.

### Artificial intelligence applications

An AI-enabled version of SCAMPs would have the potential for more rapid and robust data capture and causal inference. Early progress toward AI-informed decision-point mapping in the EHR has been undertaken for a complex SCAMP informing anticoagulation practices in children supported with extracorporeal membrane oxygenation (ECMO).^16^ A benefit of AI is the ability to ingest multimodal data (eg, EHR, text, images, waveforms from monitors, etc.) without data preprocessing that might introduce unintended bias, and to incorporate that complex data into the SCAMPs pathway. Emerging interoperability standards such as Fast Healthcare Interoperability Resources (FHIR^®^) facilitate the capture and exchange of clinician diversions from decision support to a central research community for analysis. Finally, AI-enabled causal inference algorithms allow for the rapid identification of potential data-informed modifications to the pathway in less time and with less data.

Artificial intelligence may augment SCAMPs by supporting cohort identification, data aggregation, and hypothesis generation, but introduces distinct risks. AI systems are context-bound and may amplify bias, anchor pathways to historically dominant practices, obscure rare harms, or create false confidence in recommendations.

AI should therefore be treated as a socio-technical amplifier rather than an autonomous decision-maker. Safe use requires transparency, human oversight, integration with safety governance, and mechanisms to override or suspend AI-supported recommendations when concerns arise.

### Health care payment reform

In the move from fee-for-service to value-based payment, clinician payments are being tied to improving the quality of care, reducing variation in care, and lowering costs. Accountable Care Organizations (ACOs), first launched in 2012, have become a key part of Centers for Medicare and Medicaid Services (CMS) efforts in value-based care. Analyses of the Medicare Shared Savings Program, the largest of the ACO programs, have shown improved quality as a result of efforts but inconsistent generation of cost savings; furthermore, provider attrition has remained an issue over the decade-long experience with the program.^30-32^

Within value-based payment models, including ACOs, other total cost-of-care models, and specialty-based bundle payment models, clinicians look to guidelines to assist them in efforts to improve quality and reduce variation in care. As CMS moves toward its goal of all beneficiaries being in an accountable care relationship by 2030, and as commercial payors continue to engage in more accountable care arrangements, SCAMPs have the potential to emerge as an important tool for clinicians to be successful in these value-based care payment models.^33^

From a health system leadership perspective, SCAMPs are best viewed as a potential enterprise-level framework for learning and standardization rather than isolated clinical tools. When implemented across multiple conditions, SCAMPs can align clinical governance, analytic infrastructure, and improvement strategy.

Required capabilities include clinical governance structures, analytic capacity, informatics support, and coordination with quality, safety, and equality programs. SCAMPs do not replace payer oversight or financial incentives, but may complement value-based strategies by generating internally credible, clinician-endorsed evidence on appropriateness and variation.

## International application

Although much of the published SCAMPs experience arises from the United States, the challenges they address—rising costs, workforce constraints, unwarranted variation, and limited evidence—are global.^34^ Viewed as a methodology rather than a policy instrument, SCAMPs may be relevant across diverse health systems, with adaptation to local governance, financing, and data infrastructure.

## Conclusions

SCAMPs are modifiable, rapidly learning care pathways developed by clinicians over a decade ago to address the pressing need for standardization of care and optimization of resource use. SCAMPs have demonstrated broad applicability, achievement of its aims as a quality improvement initiative, and advantages over other forms of guidelines. The experience with SCAMPs may provide a valuable paradigm on how lessons learned can lead to improvement in the content, applicability, and adoption of care guidelines. Many of the limitations of SCAMPs could be addressed with enhanced utilization of informatics tools and AI models, broadening adoption and improving iterative pathway refinement. As health care leaders look for pragmatic approaches to the incorporation of advanced clinical decision-support and AI in medicine, models such as SCAMPs merit close evaluation.

SCAMPs represent a clinician-led methodology for learning and standardization, not a guaranteed solution. Their reported successes reflect particular implementations under specific conditions. Independent evaluation, implementation science, and explicit safeguards are essential to determine when, where, and for whom SCAMPs deliver net benefit.

## Supplementary Material

qxag036_Supplementary_Data
